# Supplementary material for: Lactiplantibacillus plantarum 1201 Inhibits Intestinal Infection of Salmonella enterica subsp. enterica Serovar Typhimurium Strain ATCC 13311 in Mice with High-Fat Diet
Source: Foods. 2021 Dec 29;11(1):85. doi: 10.3390/foods11010085 (PMC8750823; doi:10.3390/foods11010085)
Supplement: Supplementary file 1 [file foods-11-00085-s001.zip › foods-1470110-supplementary.pdf]

Supporting Information

Table S1 Primer sequences for quantitative Real-Time polymerase chain reaction.

Gene		Sequence
β-actin	Forward	GCTCCTCCTGAGCGCAAGTA
	Reverse	CAGCTCAGTAACAGTCCGCC
IκB-α	Forward	GAAGAGAAGCCGCTGACCAT
	Reverse	CAGAAGTGCCTCAGCAATTCC
NF-κB	Forward	ACGATCTGTTTCCCCTCATC
	Reverse	TGCTTCTCTCCCCAGGAATA
IL-22	Forward	CCGAGGAGTCAGTGCTAAGG
	Reverse	TCTGGATGTTCTGGTCGTCA
IL-1β	Forward	ACAGGCTCCGAGATGAACAAC
	Reverse	GTGGGTGTGCCGTCTTTCAT
IL-6	Forward	CTGCAAGAGACTTCCATCCAG
	Reverse	AGTGGTATAGACAGGTCTGTTGG
IFN-γ	Forward	TGATTGCGGGGTTGTATCTG
	Reverse	CTGTCTGGCCTGCTGTTAAA
IL-17A	Forward	CTCCAGAAGGCCCTCAGACTA
	Reverse	AGCTTTCCCTCCGCATTGACA
TNF-α	Forward	CCCTCACACTCAGATCATCTTCT
	Reverse	GCTACGACGTGGGCTACAG
TGF-β1	Forward	GTCACTGGAGTTGTACGGCA
	Reverse	TCATGTCATGGATGGTGCCC
IL-10	Forward	GCTCTTACTGACTGGCATGAG
	Reverse	CGCAGCTCTAGGAGCATGTG
